# A non-endoscopic device to sample the oesophageal microbiota: a case-control study

**DOI:** 10.1016/S2468-1253(16)30086-3

**Published:** 2016-11-12

**Authors:** Daffolyn R Fels Elliott, Alan W Walker, Maria O'Donovan, Julian Parkhill, Rebecca C Fitzgerald

**Affiliations:** a Medical Research Centre Cancer Unit, Hutchison/MRC Research Centre, University of Cambridge, Cambridge, UK; b Pathogen Genomics Group, Wellcome Trust Sanger Institute, Hinxton, UK; c Rowett Institute of Nutrition and Health, University of Aberdeen, Aberdeen, UK; d Department of Histopathology, Cambridge University Hospital NHS Trust, Cambridge, UK

## Abstract

**Background:**

The strongest risk factor for oesophageal adenocarcinoma is reflux disease, and the rising incidence of this coincides with the eradication of *Helicobacter pylori*, both of which might alter the oesophageal microbiota. We aimed to profile the microbiota at different stages of Barrett's carcinogenesis and investigate the Cytosponge as a minimally invasive tool for sampling the oesophageal microbiota.

**Methods:**

In this case-control study, 16S rRNA gene amplicon sequencing was done on 210 oesophageal samples from 86 patients representing the Barrett's oesophagus progression sequence (normal squamous controls [n=20], non-dysplastic [n=24] and dysplastic Barrett's oesophagus [n=23], and oesophageal adenocarcinoma [n=19]), relevant negative controls, and replicates on the Illumina MiSeq platform. Samples were taken from patients enrolled in the BEST2 study at five UK hospitals and the OCCAMS study at six UK hospitals. We compared fresh frozen tissue, fresh frozen endoscopic brushings, and the Cytosponge device for microbial DNA yield (qPCR), diversity, and community composition.

**Findings:**

There was decreased microbial diversity in oesophageal adenocarcinoma tissue compared with tissue from healthy control patients as measured by the observed operational taxonomic unit (OTU) richness (p=0·0012), Chao estimated total richness (p=0·0004), and Shannon diversity index (p=0·0075). *Lactobacillus fermentum* was enriched in oesophageal adenocarcinoma (p=0·028), and lactic acid bacteria dominated the microenvironment in seven (47%) of 15 cases of oesophageal adenocarcinoma. Comparison of oesophageal sampling methods showed that the Cytosponge yielded more than ten-times higher quantities of microbial DNA than did endoscopic brushes or biopsies using quantitative PCR (p<0·0001). The Cytosponge samples contained the majority of taxa detected in biopsy and brush samples, but were enriched for genera from the oral cavity and stomach, including *Fusobacterium, Megasphaera, Campylobacter, Capnocytophaga*, and Dialister. The Cytosponge detected decreased microbial diversity in patients with high-grade dysplasia in comparison to control patients, as measured by the observed OTU richness (p=0·0147), Chao estimated total richness (p=0·023), and Shannon diversity index (p=0·0085).

**Interpretation:**

Alterations in microbial communities occur in the lower oesophagus in Barrett's carcinogenesis, which can be detected at the pre-invasive stage of high-grade dysplasia with the novel Cytosponge device. Our findings are potentially applicable to early disease detection, and future test development should focus on longitudinal sampling of the microbiota to monitor for changes in microbial diversity in a larger cohort of patients.

**Funding:**

Cancer Research UK, National Institute for Health Research, Medical Research Council, Wellcome Trust, The Scottish Government (RESAS).

## Introduction

Oesophageal adenocarcinoma is an aggressive malignancy with poor outcomes that generally develops from a premalignant columnar epithelium called Barrett's oesophagus. The incidence of oesophageal adenocarcinoma has increased by six times in developed countries during the past three decades.^1^ Both Barrett's oesophagus and oesophageal adenocarcinoma are thought to develop in response to chronic acid reflux in the lower oesophagus, which precipitates inflammation and mucosal injury over time.^2^ Reflux disease has increased with the obesity epidemic and altered eating habits in high-income countries, and central adiposity might also influence carcinogenesis through the release of adipokines.^3^ Additionally, epidemiological evidence suggests that the rising incidence of oesophageal adenocarcinoma coincides with the eradication of *Helicobacter pylori*, which could alter the composition of microbiota and promote bacterial overgrowth.^4^ Furthermore, reflux disease is treated with antacid drugs such as proton-pump inhibitors, which have profound effects on gastric acidity and might affect the gastro-oesophageal microbiota.^5^

There is growing evidence linking abnormal changes in the microbiota, known as dysbiosis, with human cancer. One of the best described examples is colon carcinoma, in which gastrointestinal microbiota have been shown to promote carcinogenesis in the setting of colonic inflammation.^6^, ^7^, ^8^ Studies have also linked *Fusobacterium nucleatum* to colon carcinoma through an altered tumour immuno-environment, but without associated colitis.^9^, ^10^ The oesophagus has far fewer bacteria than the colon; nevertheless, alterations in the microbiota might occur in reflux oesophagitis and Barrett's oesophagus.^11^, ^12^, ^13^ However, the role of the microbiota in Barrett's carcinogenesis is not clearly defined and there is no clinical reference standard at present for sampling the oesophageal microbiota. One of the challenges in studying the oesophageal microbiota is that endoscopy is an invasive test that provides only a focal sampling of the microbiota in biopsy samples, and a slightly larger surface area with endoscopic brushings. Minimally invasive methods for sampling the oesophageal microbiota could be clinically useful for detection and risk stratification of patients with Barrett's oesophagus.

**Research in context**

**Evidence before this study**

Epidemiological evidence suggests that the rising incidence of oesophageal adenocarcinoma coincides with the obesity epidemic, gastro-oesophageal reflux disease, and eradication of *Helicobacter pylori* with antibiotics and acid suppression treatment—all risk factors that are capable of altering the gastro-oesophageal microbiota. Three studies with small numbers of patients have shown modest alterations in the microbiota in Barrett's oesophagus and oesophagitis with 16S rRNA gene amplicon sequencing. However, studies using culture-independent methods to profile the oesophageal microbiota in oesophageal adenocarcinoma or high-grade dysplasia are lacking. One explanation for the scarcity of oesophageal microbiota studies is the challenge of endoscopic sampling and low microbial DNA yield. Based on previous studies, we postulated that the novel Cytosponge could be an effective tool to sample the microbiota along the entire length of the oesophagus.

**Added value of this study**

This study provides a comprehensive characterisation of the microbiota at different stages of the Barrett's oesophagus progression sequence using 16S rRNA gene amplicon sequencing, and compares patients with oesophageal adenocarcinoma and Barrett's oesophagus with healthy control patients. We found decreased microbial diversity in oesophageal adenocarcinoma tissue compared with controls, with enrichment of acid-tolerant bacteria such as *Lactobacillus fermentum.* The microbial diversity was reduced in the lower oesophagus regardless of whether cancerous or healthy oesophageal tissue was sampled within the same patients. We further translated our findings to the setting of early detection, using the Cytosponge to sample the microbiota in Barrett's oesophagus and high-grade dysplasia. We showed that the Cytosponge collected high microbial DNA yield and detected decreased diversity in the pre-invasive stage of high-grade dysplasia.

**Implications of all the available evidence**

Alterations in microbial communities occur in the lower oesophagus in Barrett's carcinogenesis, which are possible to detect with the minimally invasive Cytosponge. Our findings are potentially applicable to early disease detection, and future test development should focus on longitudinal sampling of the microbiota to monitor for changes in microbial diversity in a larger cohort of patients.

Here we investigate the Cytosponge prototype (Europlaz, Southminster, UK) as a non-endoscopic cell-sampling device that can collect a representative sample of cells along the length of the oesophagus.^14^, ^15^ The device consists of a spherical mesh that is compressed within a gelatine capsule and attached to a string. Once swallowed, the capsule dissolves and the Cytosponge expands in the patient's stomach before being withdrawn on a string through the patient's mouth. We have previously shown that this device is a safe, acceptable method for diagnosing Barrett's oesophagus, with promising accuracy and cost-effectiveness.^14^, ^15^, ^16^ The goal of this study was to provide a comprehensive description of the microbiota in the different pathogenic stages of oesophageal adenocarcinoma using 16S rRNA gene amplicon sequencing and to test the feasibility of the Cytosponge to detect changes in the microbiota occurring in Barrett's oesophagus and high-grade dysplasia.

## Methods

### Study design and participants

In this case-control study, endoscopic biopsies, brushes, Cytosponge samples, and throat swabs were collected from patients with a diagnosis of non-dysplastic Barrett's oesophagus or high-grade dysplasia, and from control patients with symptoms of reflux or dyspepsia enrolled in the Barrett's Oesophagus Screening Trial (BEST2) at five UK hospitals. In patients with a diagnosis of oesophageal adenocarcinoma, tissue samples from the tumour and matched normal squamous oesophagus were collected from six UK hospitals participating in Oesophageal Cancer Clinical and Molecular Stratification (OCCAMS) for the International Cancer Genome Consortium (ICGC).

The patient inclusion criteria included age between 20 and 90 years with either normal endoscopy or endoscopic and histological documentation of Barrett's oesophagus or oesophageal adenocarcinoma. The exclusion criteria were current infection, recent antibiotic treatment, previous chemotherapy treatment, and documented pathological findings unrelated to Barrett's oesophagus or oesophageal adenocarcinoma. Ethical approval was obtained from the National Research Ethics Services Cambridgeshire Research Ethics Committee on behalf of all hospital centres in the BEST2 trial (REC 10/H0308/71) and the OCCAMS/ICGC trial (REC 07/H0305/52 and 10/H0305/51). Written informed consent was obtained from all participants before the collection of samples and recording of clinical information.

### Procedures

For patients enrolled in the BEST2 study, matched endoscopic biopsies were taken from an area of Barrett's oesophagus and proximal normal squamous oesophagus. Endoscopic brushings were taken from an area of normal squamous oesophagus only. The tissue samples collected in the OCCAMS/ICGC study included endoscopic biopsies, endoscopic mucosal resection specimens, and surgical biopsies after oesophagectomy. Sampling from oesophagectomy specimens was done with a sterile scalpel blade (cutting down to submucosa) within 1 h of surgical resection. All samples were flash frozen in liquid nitrogen and stored at −80°C except for the Cytosponge samples, which were preserved in BD SurePath liquid at 4°C. All patients fasted overnight before endoscopy or surgery. As part of routine perioperative procedure, patients with oesophageal adenocarcinoma who underwent oesophagectomy received prophylactic intravenous antibiotics at the time of surgery, up to 6 h before the research samples were obtained. The very close timing of this perioperative antibiotic exposure should not greatly affect microbial community composition profiles because 16S rRNA gene amplicon sequencing detects both live and dead bacterial cells cross-sectionally (appendix p 1).^17^

Cytosponge samples were vortexed and centrifuged to pellet cellular debris (215 g for 5 min), and the residual supernatant was used for microbial DNA extraction after further high-speed centrifugation (14 000 g for 10 min). DNA was isolated from all oesophageal samples using the Precellys Soil DNA Kit (Peqlab, Southampton, UK). The 16S rRNA gene was amplified using primers for the V1-V2 region: 27F 5′AATGATACGGCGACCACCGAGATCTACAC TATGGTAATT CC AGMGTTYGATYMTGGCTCAG and 338R 5′CAAGCAGAAGACGGCATACGAGAT NNNNNNNNNNNN AGTCAGTCAG AA GCTGCCTCCCGTAGGAGT, where Illumina adapter sequences are at the 5′ end, and the N string is a unique barcode. Most samples had two barcoded replicates to ensure reproducibility (labelled A or B in the appendix p 5), and negative controls from every DNA extraction step underwent additional PCR cycles to identify contaminant organisms. The reaction conditions were 98°C for 2 min, 25 cycles at 98°C for 30 s, 50°C for 30 s, and 72°C for 90 s, and extension at 72°C for 5 min. Negative controls were kit reagents or nuclease-free water, which underwent 45 amplification cycles. All samples were amplified in duplicate and pooled to minimise PCR bias and maximise yield. The PCR products were concentrated using ethanol precipitation and quantified using a Qubit 2·0 Fluorometer before sequencing on the MiSeq Illumina platform using 2 × 250 bp read length. 16S rRNA gene amplicon sequencing data have been deposited in the European Nucleotide Archive under accession number ERP005191. The 16S rRNA gene amplicon sequence analysis was done with mothur.^18^ The MiSeq standard operating procedure^19^ was followed with the exception of chimera checking, which was done with chimera.perseus, and unique sequences were removed using the split.abund and remove.seqs commands before building the distance matrix. Contaminant operational taxonomic units (OTUs) were defined as having greater proportional abundance in negative controls, alongside previous evidence that these OTUs were derived from genera that are common contaminants,^20^ and these reads were removed using remove.seqs (3434 OTUs defined as contaminants from 5757 total OTUs). Comparisons between replicate samples (using Metastats^21^ as implemented in mothur) showed no significant differences so replicates were pooled using the Linux sed command to maximise the number of reads per sample, and samples with fewer than 550 reads or Good's coverage less than 95% were removed with remove.groups. The Good's coverage estimator^22^ is used to assess what proportion of the total OTUs present within a given sample are detected in the sequencing results, and thus gives an indication of how thorough the sampling has been at the chosen sequencing depth. For measures of diversity that are sensitive to the sequencing depth, random subsampling was done at the lowest number of reads per sample using the sub.sample command in mothur. Data were subsampled at 631 reads for the analysis of tissue samples (median Good's coverage 96·36%, range 92·08–99·37), 656 reads for matched tumour-normal pairs (97·41%, range 95·27–98·78), 631 reads for the comparison of different sampling methods (96·51%, range 91·13–99·84), and 19 303 reads for the analysis of Cytosponge samples (99·83%, range 99·70–99·95). When determining the shared genera between Cytosponge samples, biopsies, and brushes, a cutoff of 0·0001% proportional abundance was used to focus on the more abundant OTUs that are less likely to be susceptible to errors introduced by subsampling (below 0·0001% there were less than 27 reads supporting each OTU across all the samples). A second cutoff value of 0·1% was chosen arbitrarily to show the similarity between sample types and is a common cutoff used in previous studies.^23^, ^24^ Sequence identity was confirmed at the species level, where possible, by carrying out NCBI BLAST analysis on representative sequences using MegaBLAST.^25^

Quantitative PCR for the 16S rRNA gene was done with SYBR Green I Master Mix (Roche, Mannheim, Germany) on the LC480 LightCycler 480 II (Roche, Mannheim, Germany), in triplicate. The reaction conditions were 95°C for 5 min, 40 cycles at 95°C for 15 s, 60°C for 30 s, and 72°C for 90 s, and a melt curve. The primer sequences were 331F 5′TCCTACGGGAGGCAGCAGT and 797R 5′GGACTACCAGGGTATCTAATCCTGTT.^26^

### Statistical analysis

The non-parametric Kruskal-Wallis test and Dunns multiple comparisons post-test were used for comparisons between diagnostic groups in Graphpad Prism (version 6). Within Graphpad, the Wilcoxon signed rank test was used for analyses involving matched tumour-normal pairs, and the Friedman test and Dunns post-test were used for analyses of matched samples from endoscopic biopsies, brushes, and the Cytosponge. We used LEfSe,^27^ a metagenomic biomarker discovery method, to identify microbial taxa that differed significantly between controls, Barrett's oesophagus, and oesophageal adenocarcinoma samples. LEfSe was used within mothur. All sequencing reads were included for the composition analysis using LEfSe. LEfSe ranks OTUs in the order that it considers these taxa to be most likely to explain differences between microbial communities using linear discriminant analysis to estimate effect size. A full explanation of the statistical approaches used in LEfSe can be found in the original article by Segata and colleagues.^27^ Within mothur, the Bray-Curtis calculator was used to describe the dissimilarity between communities by taking into account both the overlap in OTUs that are present between samples and the proportional abundance of those OTUs in each sample. Using dissimilarity information calculated with the Bray-Curtis calculator, the parsimony test and the analysis of molecular variance (AMOVA) test were used to show significantly different clustering between microbiota profiles from the different diagnostic groups. IBM SPSS Statistics (version 24) was used to analyse patient data (ANOVA for mean age and Fisher's exact test for sex, ethnicity, and antacid usage). A significant p value was defined as less than 0·05.

### Role of the funding source

The funders had no role in the study design, data analysis, interpretation of data, or writing of the report. The corresponding author had full access to all the data in the study and had final responsibility for the decision to submit for publication.

## Results

To investigate whether the development of oesophageal adenocarcinoma was associated with dysbiosis, we did 16S rRNA gene amplicon sequencing on tissue samples from patients with oesophageal adenocarcinoma (n=19), Barrett's oesophagus (n=24), and healthy control patients (n=19; table). Patients with oesophageal adenocarcinoma and Barrett's oesophagus were older (p=0·001) and predominantly male (p=0·025) in comparison with the control patients, which is consistent with the known epidemiology of this disease.^28^ The number of patients recruited at each participating hospital centre and additional clinicopathological data for patients with oesophageal adenocarcinoma is provided in the appendix (pp 3, 4). Acid-suppressant drugs were taken regularly by 23 (100%) of 23 patients with dysplasia, 22 (92%) of 24 patients with Barrett's oesophagus, 12 (71%) of 17 patients with oesophageal adenocarcinoma (two did not report whether or not they were taking acid-suppressant drugs), and 15 (75%) of 20 control patients. One patient with Barrett's oesophagus and two patients with high-grade dysplasia reported taking a course of antibiotics within the past month.

**Table:** Patient demographics and oesophageal samples

		**Control (n=20)**	**Non-dysplastic Barrett's oesophagus (n=24)**	**High-grade dysplasia (n=23)**	**Oesophageal adenocarcinoma (n=19)**	**p value**
Age, years		57 (29–86)	68 (53–79)	65 (50–82)	70 (44–79)	0·001
Sex						
	Male	7 (35%)	16 (67%)	19 (83%)	15 (79%)	0·006
	Female	13 (65%)	8 (33%)	4 (17%)	4 (21%)	..
White ethnicity		19 (95%)	24 (100%)	23 (100%)	16 (89%)*	0·089
Antacid usage		15 (75%)	22 (92%)	23 (100%)	12 (71%)*	0·011
Samples passing quality control/samples sequenced						
	Cytosponge	20/20	24/24	23/23		..
	Brush (squamous only)	19/19	19/19	..	..	..
	Tissue	16/19	17/24 (Barrett's oesophagus); 15/24 (squamous)†	..	15/19 (oesophageal adenocarcinoma); 15/19 (squamous)†	..
Total number of samples sequenced		58	91	23	38	..

Data are n (%) or median (range) unless otherwise stated. ..=samples were not sequenced for all diagnostic categories for the following reasons: it was not safe or appropriate to have patients with cancer swallow the Cytosponge; we compared brush sampling methods for areas of normal oesophagus in patients with no pathology or Barrett's oesophagus only; and tissue samples were not available for dysplasia.

† Tissue samples from the area of Barrett's oesophagus or tumour and matched normal squamous oesophagus were taken in the same cases.

* Not recorded, ethnicity unknown for one patient and antacid usage unknown for two patients.

After filtering the sequencing data and removing contaminant sequences, the mean number of reads for tissue samples was 6649 (SD 10421) and the median was 3064 (5688) and the proportion of reads that were subsampled for diversity analyses was 9·5% (for 631 reads cutoff) and 9·9% (for 656 reads cutoff). 14 tissue samples did not meet quality criteria and were excluded from the analysis, leaving 16 control, 17 Barrett's oesophagus, and 15 oesophageal adenocarcinoma samples. Five phyla accounted for the majority of sequencing reads in the dataset: Firmicutes (59·9%), Bacteroidetes (15·1%), Proteobacteria (12·8%), Actinobacteria (5·8%), and Fusobacteria (5·4%). 1060 OTUs were identified and classified as belonging to 345 different genera.

**Figure 1:**
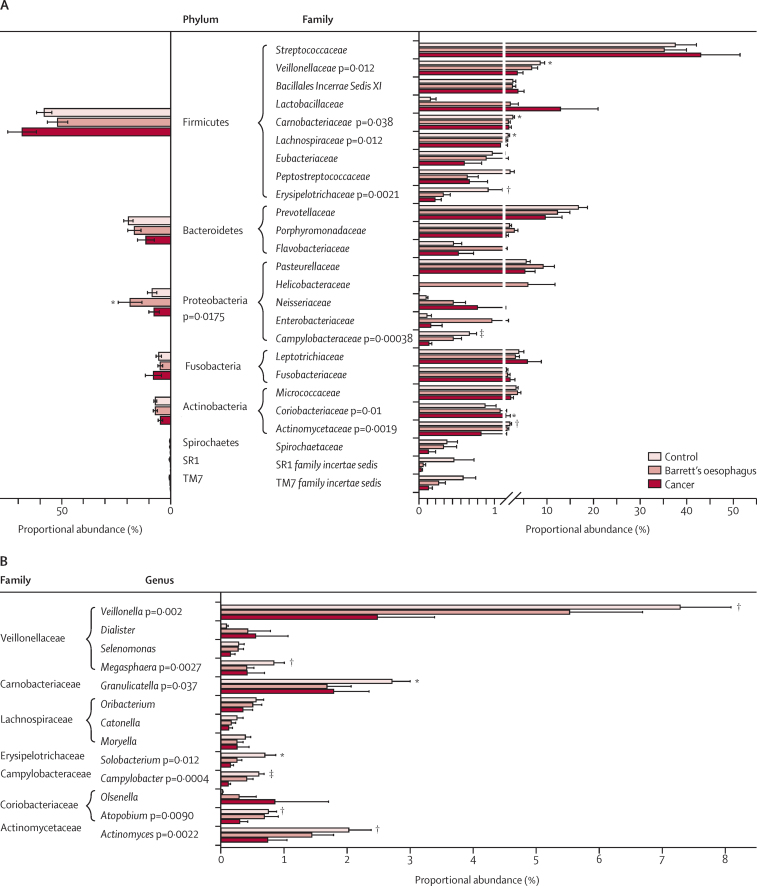
Proportional abundance of microbial taxa (A) Mean proportional abundance of the eight most prevalent phyla and 25 most prevalent families in tissue samples for healthy control patients (n=16), patients with Barrett's oesophagus (n=17), and patients with oesophageal adenocarcinoma (n=15). Significant differences were calculated with linear discriminant analysis effect size (LEfSe), and error bars are standard error of the mean. (B) Mean proportional abundance of representative genera from significantly enriched families identified in (A). Only genera with overall proportional abundances greater than 0·1% are included and error bars are standard error of the mean. *p<0·05. †p<0·01. ‡p<0·001.

By LEfSe, at the phylum level, the Barrett's samples contained a higher proportional abundance of Proteobacteria (mean 18·6%, median 14·8%, SE 5·5%) compared with controls (mean 8·5%, median 8·2%, SE 5·5%; p=0·017) and oesophageal adenocarcinoma samples (mean 7·6%, median 3·9%, SE 2·4%). The control samples were enriched for several taxa at the family level, including the Gram-negative, anaerobic Veillonellaceae (p=0·012, overall proportional abundance 5·3%) and microaerophilic Campylobacteraceae (p=0·00038, overall proportional abundance 0·2%), the Gram-positive, anaerobic Lachnospiraceae (p=0·012, overall proportional abundance 1%) and Erysipelotrichaceae (p=0·0021, overall proportional abundance 0·4%), and the Gram-positive, facultative anaerobic Carnobacteriaceae (p=0·038, overall proportional abundance 1·6%), and Actinomycetaceae (p=0·0019, overall proportional abundance 0·8%; figure 1A). Significant genera within these families included *Veillonella* (p=0·002, overall proportional abundance 3·8%), *Megasphaera* (p=0·0027, overall proportional abundance 0·3%), *Granulicatella* (p=0·037, overall proportional abundance 1·6%), *Actinomyces* (p=0·0022, overall proportional abundance 0·8%), *Solobacterium* (p=0·012, overall proportional abundance 0·3%), and *Campylobacter* (p=0·0004, overall proportional abundance 0·2%; figure 1B). In oesophageal adenocarcinoma samples, the Gram-positive, anaerobic Coriobacteriaceae was enriched at the family level (p=0·01, overall proportional abundance 1·9%), but there were no significant genera identified within this family. At the species level, the Gram-positive, facultative anaerobic *Lactobacillus fermentum* was enriched in oesophageal adenocarcinoma with mean proportional abundance of 0·6% (median 0·009%, SE 0·5%) compared with 0·01% (median 0%, SE 0·007%) in Barrett's oesophagus samples and 0·004% (median 0%, SE 0·003%) in control samples (p=0·028). Sequence identity was confirmed where possible with NCBI BLAST. One Barrett's oesophagus sample contained a high proportional abundance of *H pylori* sequences (>99%).

**Figure 2:**
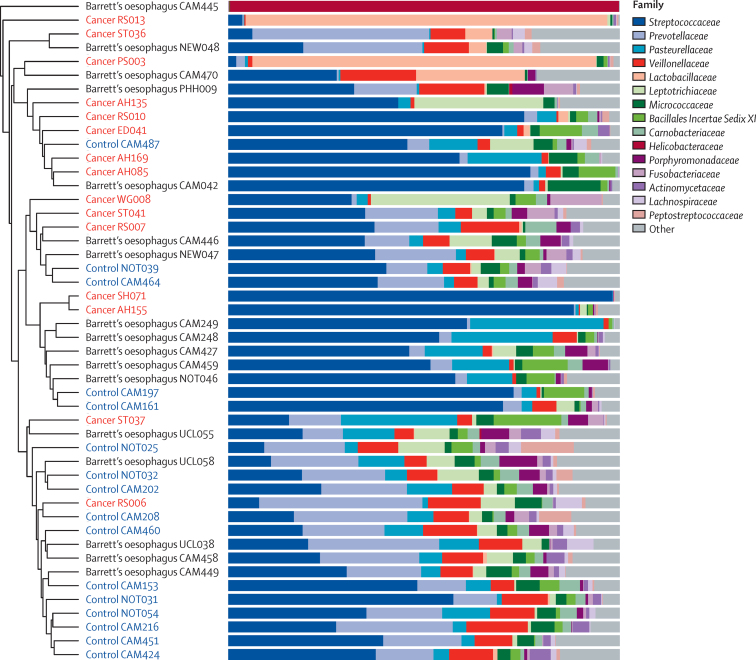
Microbial community composition in Barrett's oesophagus The oesophageal adenocarcinoma and control patient groups largely cluster away from each other in this Bray-Curtis cluster dendrogram (p=0·002, parsimony test), but there is no significant difference in clustering for Barrett's oesophagus. Microbial composition is shown at the family level for each tissue sample. Data were sub-sampled at 631 reads per sample.

Typically, oesophageal adenocarcinoma samples clustered away from controls in a Bray-Curtis cluster dendrogram (p=0·002, parsimony test), emphasising the difference in community structure (figure 2). The Bray-Curtis algorithm describes the dissimilarity between communities by taking into account both the overlap in OTUs that are present and the proportional abundance of those OTUs. Samples that have fewer overlapping OTUs and OTUs with less similar proportional abundances will cluster separately, and this differential clustering was further shown by principal coordinate analysis (p=0·001, AMOVA test; appendix p 1). The microbial communities of seven of 15 oesophageal adenocarcinoma samples were dominated by the Gram-positive order Lactobacillales. Of the seven patients with a high proportion of acid-tolerant Lactobacillales, six were taking antacid drugs. Five oesophageal adenocarcinoma samples had a high proportional abundance of *Streptococcus* spp (69–98%) and two samples had a high proportional abundance of *Lactobacillus* spp (87% and 92%). NCBI BLAST showed that the representative species were *Streptococcus pneumoniae*/*mitis, Streptococcus salivarius/vestibularis, Streptococcus parasanguinis, Lactobacillus gasseri, and Lactobacillus helveticus*/*suntoryeus/gallinarum* (/ indicates where it was not possible to differentiate between different species using the 16S rRNA gene regions sequenced). Although abundant in the acidic stomach environment, such a high proportional abundance of *Lactobacillus* spp was an unexpected finding in the oesophagus. When we examined matched healthy and tumour tissue for the two patients with high *Lactobacillus* spp, we found that this genus dominated the lower oesophagus regardless of disease state (appendix p 2). Gram-positive rods were visualised in areas of ulceration in tumour PS003 with a high proportional abundance of *Lactobacillus* spp (appendix p 2).

**Figure 3:**
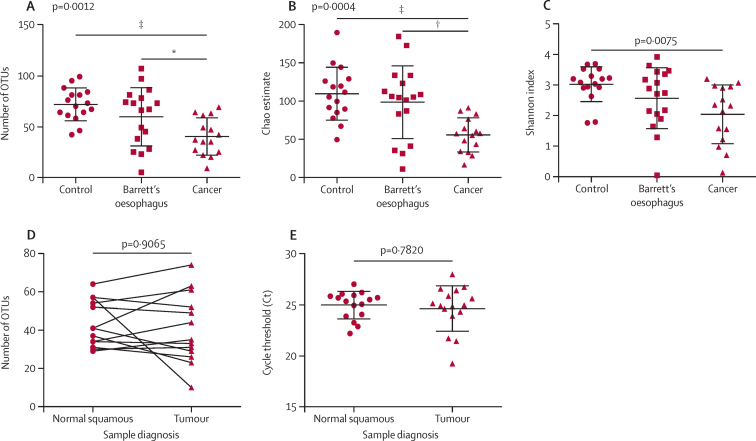
Microbial alpha diversity in oesophageal adenocarcinoma (A) Observed richness of bacterial operational taxonomic units (OTUs). (B) The Chao estimate of total OTU richness and (C) the Shannon diversity index are shown for tissue samples from healthy control patients (n=16), patients with Barrett's oesophagus (n=17), and patients with oesophageal adenocarcinoma (n=15). Statistical significance was calculated with the Kruskal-Wallis test and Dunns multiple comparisons post test. Data were subsampled at 631 reads per sample. (D) Observed richness of bacterial OTUs for paired normal squamous and tumour tissue samples from 13 patients (26 samples), Wilcoxon signed rank test. Data were subsampled at 656 reads per sample. (E) Overall bacterial abundance using 16S rRNA gene qPCR in matched tumour and normal squamous tissue from 16 patients (32 samples), Wilcoxon signed rank test. Error bars represent standard deviation. *p<0·05. †p<0·01. ‡p<0·001.

Alpha diversity refers to the species diversity within a given environment and includes the number of species (richness) and the proportion of those species (evenness) within the microbial community. Three indices of alpha diversity, observed OTU richness (p=0·0012; figure 3A), the Chao estimate of total OTU richness (p=0·0004; figure 3B), and the Shannon diversity index (p=0·0075; figure 3C) showed that diversity was lower in oesophageal adenocarcinoma samples than in controls. In comparison with Barrett's oesophagus samples, the oesophageal adenocarcinoma samples showed a decrease in observed OTU richness and the Chao estimate, but not in the Shannon diversity index. 13 patients with oesophageal adenocarcinoma had matched normal squamous tissue sampled proximal to the tumour, and in these patients there was no difference in OTU richness between the normal and tumour tissue (p=0·9065; figure 3D). Similarly, there was no difference for the Chao estimate (p>0·999) or the Shannon index (p=0·6355). Furthermore, there was no difference in overall bacterial abundance between matched normal squamous and tumour tissue (p=0·782; figure 3E). These results suggested that the decreased microbial diversity was pervasive throughout the lower oesophagus in oesophageal adenocarcinoma and was independent of the absolute quantity of oesophageal bacteria.

We did additional analyses to ensure that the differences noted were not due to sex, age, or acid suppression. We repeated the diversity analysis with male patients only (excluding ten control patients, four patients with Barrett's oesophagus, and three patients with cancer) and the results were consistent, with decreases in diversity in cancer samples compared with controls for the observed OTU richness (p=0·0029), the Chao estimate of total OTU richness (p=0·0017), and the Shannon diversity index (p=0·0070). We repeated the diversity analysis with patients aged 60 years and older (excluding nine control patients, two patients with Barrett's oesophagus, and two patients with cancer) and the results showed a similar trend, which was significant for the observed OTU richness (p=0·0448), the Chao estimate of total OTU richness (p=0·0288), but not the Shannon diversity index (p=0·0892). We also did a subgroup analysis for age within each diagnostic subgroup (using median age as a cutoff within each group) and there were no significant differences in diversity for younger versus older patients within any of the subgroups. When we excluded patients who were not taking acid suppression (four control patients, two patients with Barrett's oesphagus, four patients with cancer) or unknown acid suppression status (one patient with cancer) the results were similar, with decreased diversity in oesophageal adenocarcinoma samples compared with controls, as evidenced by the observed OTU richness (p=0·0065), the Chao estimate of total OTU richness (p=0·0033), and the Shannon diversity index (p=0·0202).

Given that the decrease in microbial diversity in oesophageal adenocarcinoma seemed widespread throughout the oesophagus, we questioned whether the Cytosponge could be a useful tool to sample the microbiota along the entire length of the oesophagus and upper gastrointestinal tract. 15 patients with Barrett's oesophagus and 16 control patients had Cytosponge samples that underwent 16S rRNA gene amplicon sequencing with matched endoscopic biopsies and brushes taken from an area of normal squamous oesophagus. 13 of these patients also had swabs of their posterior pharynx to analyse the similarities and differences between the oesophageal and oral microbiota. Overall, 1455 OTUs were identified and mapped to 381 genera. Using a cutoff of 0·0001% overall proportional abundance (138 genera classified), 84·1% of genera were shared between Cytosponge samples, biopsies, and brushes, and 83·6% were shared between Cytosponge samples and throat swabs. A stricter cutoff of 0·1% overall proportional abundance (41 genera classified) showed supporting reads for 100% of genera in all sample types, suggesting an overlap in community membership between the oral cavity, oesophagus, and gastric cardia.

**Figure 4:**
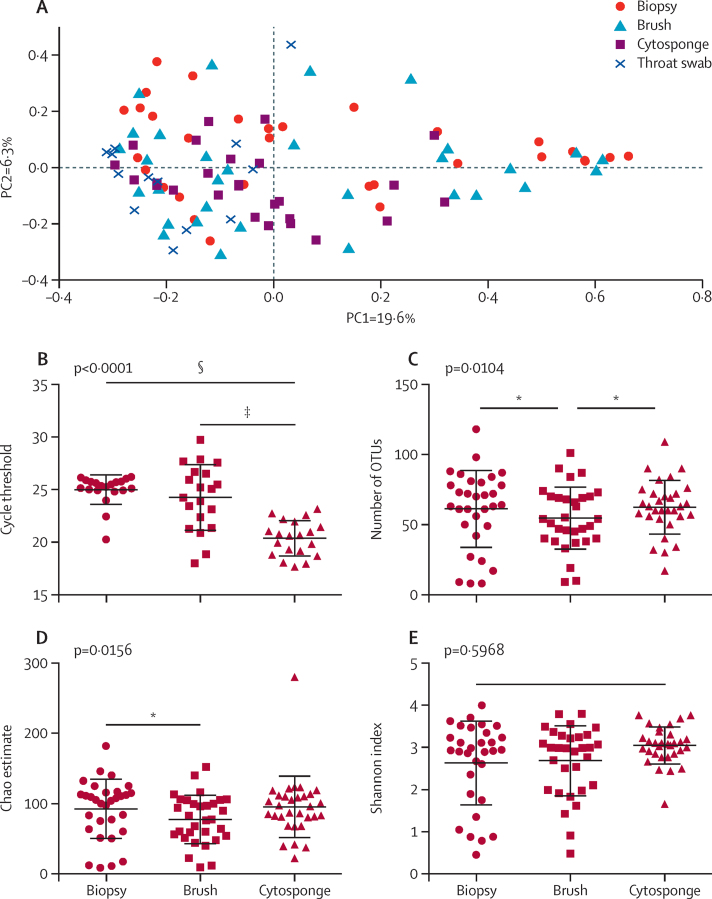
Comparison of different methods to sample the oesophageal microbiota (A) Principal coordinate analysis with the Bray-Curtis algorithm for matched endoscopic biopsies, brushes, and Cytosponge samples (31 patients) and 13 throat swabs from a subset of these patients. The first axis (PC1) accounts for 19·6% of the sample variance and the second axis (PC2) accounts for 6·3% of the variance. Data were subsampled at 631 reads per sample. (B) Overall bacterial abundance using 16S rRNA gene-based quantitative PCR in matched endoscopic biopsies, brushes, and Cytosponge samples (20 patients), Friedman test and Dunns multiple comparisons post test. (C) The observed diversity of bacterial operational taxonomic units (OTUs), (D) the Chao estimate of total OTU richness, and (E) the Shannon diversity index for matched endoscopic biopsies, brushes, and Cytosponge samples (31 patients), Friedman test and Dunns multiple comparisons post test. Data were subsampled at 631 reads per sample. *p<0·05. †p<0·01. ‡p<0·001. §p<0·0001.

Although most microbial taxa overlapped between sample types, the proportional abundances differed. At the phylum level, the Cytosponge samples contained a higher proportional abundance of Tenericutes in comparison with the other sample types using LEfSe (p = 4·7 × 10^−5^, overall proportional abundance 0·2%). At the genus level, the Cytosponge samples contained greater proportional abundances of *Fusobacterium* (p<0·0001, overall proportional abundance 2%), *Megasphaera* (p<0·0001, overall proportional abundance 1·8%), *Campylobacter* (p<0·0001, overall proportional abundance 1·7%), *Capnocytophaga* (p=0·00058, overall proportional abundance 0·7%), and *Dialister* (p<0·0001, overall proportional abundance 0·2%). In keeping with these findings, principal coordinate analysis with the Bray-Curtis algorithm showed that the Cytosponge samples clustered away from the throat swabs, endoscopic biopsies, and brushes (p<0·001, AMOVA test; figure 4A). The throat swabs clustered distinctly from all the other sample types as well (p<0·001). There was no difference in clustering between biopsies and brushes on the principal coordinate analysis plot (p=0·459).

As expected, because of increased sampling surface area, quantitative PCR of overall bacterial abundance showed the quantity of microbial DNA isolated from Cytosponge samples was greater than that from matched biopsies and brushes (20 patients, p<0·0001; figure 4B). After subsampling to normalise for sequencing depth, there was a decrease in observed OTU richness (p=0·0104; figure 4C) and the Chao estimate of total OTU richness (p=0·0156; figure 4D) in endoscopic brush samples, but no difference for the Shannon index (p=0·5968; figure 4E).

**Figure 5:**
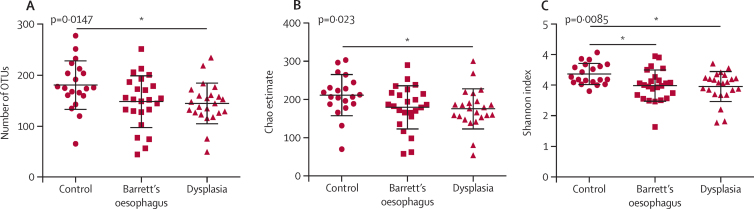
Microbial alpha diversity in high-grade dysplasia detected with the Cytosponge (A) Observed richness of bacterial operational taxonomic units (OTUs), (B) the Chao estimate of total OTU richness, and (C) the Shannon diversity index for Cytosponge samples taken from normal squamous control patients (n=20), patients with Barrett's oesophagus (n=24), and patients with high-grade dysplasia (n=23). Statistical significance was calculated with the Kruskal-Wallis test and Dunns multiple comparisons post-test. Data were sub-sampled at 19 303 reads per sample. Error bars represent standard deviation. *p<0·05.

To translate our findings to the setting of early detection, we tested the usefulness of the Cytosponge to detect changes in microbial diversity in patients with high-grade dysplasia (n=23). The mean number of reads for Cytosponge samples was 40 753 (SD 9717) and the median was 40 821 (11714), and the proportion of reads that were subsampled was 47% (for 19 303 reads cutoff). The observed OTU richness was decreased in high-grade dysplasia compared with controls (p=0·0147; figure 5A), as were the Chao estimate of total OTU richness (p=0·023; figure 5B) and the Shannon index (p=0·0085; figure 5C). There was decreased diversity in Barrett's oesophagus, but this was significant only for the Shannon index. In general, the Cytosponge samples showed homogeneous results for microbiota composition between diagnostic groups at the phylum and family levels, suggesting that the fraction of microbiota sampled from the area of Barrett's oesophagus was diluted by the copious bacteria sampled from the rest of the oesophagus, oral cavity, and stomach. Despite this, three genera were identified that distinguished controls from the other sample types using LEfSe: *Dialister* (p=0·027, overall proportional abundance 0·3%), *Schlegelella* (p=0·016, overall proportional abundance 0·1%), and unclassified *Prevotellaceae* (p=0·047, overall proportional abundance 1·3%).

## Discussion

Our sequencing data showed decreased microbial diversity and altered community composition in oesophageal adenocarcinoma. Interestingly, patients with oesophageal adenocarcinoma appeared to have this reduced diversity regardless of whether cancerous or normal oesophageal tissue was sampled. The genera that were decreased in proportional abundance in oesophageal adenocarcinoma included Gram-negative (*Veillonella, Megasphaera*, and *Campylobacter*) and Gram-positive taxa (*Granulicatella, Atopobium, Actinomyces*, and *Solobacterium*). There was significantly increased proportional abundance for *L fermentum* in patients with oesophageal adenocarcinoma compared with control patients and those with Barrett's oesophagus, and there was a high proportional abundance of acid-tolerant Lactobacillales (*Lactobacillus* spp and *Streptococcus* spp) in a subset (seven [47%] of 15) of oesophageal adenocarcinoma samples. To translate our findings to the setting of early detection, we investigated the use of the Cytosponge device for sampling the oesophageal microbiota in Barrett's oesophagus and high-grade dysplasia. The Cytosponge had high microbial DNA yield and detected significantly decreased diversity in patients with high-grade dysplasia compared with control patients.

Lactobacillales, which are lactic acid bacteria, are so named for their ability to produce lactate from the fermentation of carbohydrates and to survive under harsh acidic conditions.^29^ Their resilience to low pH might enable *Lactobacillus* spp and *Streptococcus* spp to thrive in the tumour niche in a subset of patients with oesophageal adenocarcinoma, and production of lactic acid by these bacteria could further acidify the microenvironment. Lactic acid fermentation can also produce noxious by-products, such as hydrogen peroxide, that directly inhibit the growth of competitor bacteria and enable Lactobacillales to dominate the lower oesophagus. Given the altered microbial composition in oesophageal adenocarcinoma samples, it would be interesting to correlate microbiota data with expression and activity of Toll-like receptors (TLRs), particularly TLR2, given the increased proportional abundance of Gram-positive genera in a subset of cancer samples. Other authors have investigated TLR expression in Barrett's carcinogenesis and found overexpression of TLRs 1, 2, 4, 6, and 9 in human oesophageal adenocarcinoma samples^30^, ^31^, ^32^, ^33^ and TLRs 1–3, 6, 7, and 9 in a rat reflux model.^34^

Although the microbial community structure differed significantly in oesophageal adenocarcinoma in our study, there was only a modest reduction in diversity in Barrett's oesophagus and no genera were identified that discriminated between controls and Barrett's oesophagus, or between Barrett's oesophagus and oesophageal adenocarcinoma. It is possible that very low abundance genera might be difficult to detect in oesophageal biopsies given the low microbial DNA yield, and notably some pathogens have been shown to cause overt disease while only accounting for a low proportional abundance of the total microbiota, such as *Clostridium difficile*,^35^ *Citrobacter rodentium*,^36^ and *Fusobacterium* spp.^37^ Similarly, Amir and colleagues^5^ were unable to identify any taxa that differentiated between controls (n=15) and Barrett's oesophagus (n=6), or oesophagitis (n=13) using LEfSe. By contrast, Yang and colleagues^11^ reported that Gram-negative bacteria were significantly enriched in Barrett's oesophagus (n=10) and reflux oesophagitis (n=12) compared with controls (n=12).^11^ The main limiting factor of these microbiota studies is the relatively small sample size and substantial inter-individual variation in microbiota composition. Another limitation is that although LEfSe is useful for biological interpretation of metagenomic data, it does not correct for multiple comparisons, so there is a risk of false discovery (p value, α=0·05). The inclusion of appropriate negative controls and replicate samples is also paramount for low microbial biomass samples to facilitate removal of contaminant OTUs that might also lead to false discovery,^20^ and this was a major strength of our study. We also imposed strict quality control criteria, resulting in the exclusion of 14 tissue samples with low sequencing read numbers and Good's coverage estimates. The difficulty in obtaining good quality sequencing data from oesophageal samples highlights the potential use of the Cytosponge device, which samples a larger surface area.

Our results suggest that it is feasible to sample oesophageal microbiota using the Cytosponge, and the device detected the majority of genera present in endoscopic biopsies and brushes. The high microbial DNA yield collected by the Cytosponge reflects sampling of the entire length of the oesophagus as well as the proximal stomach and oral cavity as it is withdrawn. The throat swabs showed similarities in community membership between the oral cavity and oesophagus, but the proportional abundances differed, as shown by distinct clustering in principal coordinate analysis. Despite dilution from sampling the upper gastrointestinal tract, it was still possible for the Cytosponge to detect a decrease in diversity and community composition between normal squamous controls and high-grade dysplasia. Similar to the Cytosponge, Fillon and colleagues^38^ described a minimally invasive oesophageal string test to sample the microbiota in a paediatric population. The oesophageal string test detected a similar microbial composition to that in matched oesophageal biopsies, but required the patients to remain in hospital overnight with the string secured to their cheek. Alternatively, the Cytosponge is a convenient test that can be given in a general practitioner's or family doctor's office with the supervision of a trained nurse, and takes only 5–7 min to complete.^14^, ^39^ The Cytosponge can also provide histological data for inflammatory pathologies such as candidal oesophagitis, herpes oesophagitis, and eosinophilic oesophagitis.^40^ Our initial results using the Cytosponge are promising, and future test development should focus on longitudinal sampling of the microbiota to monitor changes in microbial diversity over time in a larger cohort of patients. Further research should also examine the role of diet, dysphagia, and other external influences on the oesophageal microbiota.

**This online publication has been corrected twice. The first corrected version appeared at thelancet.com/gastrohep on December 9, 2016, and the second on October 4, 2017**
