# Correction to *Lancet Gastroenterol Hepatol* 2017; 2: 32–42

**DOI:** 10.1016/S2468-1253(16)30188-1

**Published:** 2016-12-10

*Fels Elliott DR, Walker AW, O'Donovan M, Parkhill J, Fitzgerald RC. Use of a non-endoscopic device to sample the oesophageal microbiota: a case-control study.* Lancet Gastroenterol Hepatol *2017; **2:** 32–42*—In the legend of figure 4 of this Article, the footnotes should read “*p<0·05. †p<0·01. ‡p<0·001. §p<0·0001.” This correction has been made to the online version as of Dec 9, 2016, and the printed version is correct.

