# Correction to *Lancet Gastroenterol Hepatol* 2017; 2: 32–42

**DOI:** 10.1016/S2468-1253(17)30299-6

**Published:** 2017-10-04

*Fels Elliott DR, Walker AW, O'Donovan M, Parkhill J, Fitzgerald RC. A non-endoscopic device to sample the oesophageal microbiota: a case-control study.* Lancet Gastroenterol Hepatol *2017; **2:** 32–42*—This Article is an Open Access article under the CC BY 4.0 license. Copyright belongs to the authors. This correction has been made to the online version as of Oct 4, 2017.

