# Supplementary material for: A non-endoscopic device to sample the oesophageal microbiota: a case-control study
Source: Lancet Gastroenterol Hepatol. 2016 Nov 12;2(1):32–42. doi: 10.1016/S2468-1253(16)30086-3 (PMC5656094; doi:10.1016/S2468-1253(16)30086-3)
Supplement: Supplementary appendix [file mmc1.pdf]

THE LANCET
Gastroenterology & Hepatology

Supplementary appendix

This appendix formed part of the original submission and has been peer reviewed. We post it as supplied by the authors.

Supplement to: Elliott DRF, Walker AW, O'Donovan M, Parkhill J, Fitzgerald RC. A non-endoscopic device to sample the oesophageal microbiota: a case-control study. *Lancet Gastroenterol Hepatol* 2016; published online Nov 11. http://dx.doi.org/10.1016/S2468-1253(16)30086-3.

Web Appendix

Supplementary Text

Patients who underwent oesophagectomy received perioperative cefazoline, a cephalosporin antibiotic that is bactericidal and interferes with cell wall synthesis. Although the bacteria undergo cell lysis, which could lead to loss of nuclear material, the oesophageal tissue samples were taken immediately after the oesophagus was surgically resected and snap frozen in liquid nitrogen. We expect that this short time interval should minimise the opportunity for DNA to be washed away (notably the patient does not swallow saliva while they are paralysed for the procedure). In support of this, there were no significant differences in alpha diversity measures between tumour samples that underwent surgery and received preoperative antibiotics compared to tumour samples collected by endoscopic biopsy. Furthermore, after excluding the six OAC patients who took antibiotics from our diversity analysis, the trend was the same and showed decrease diversity in cancer samples compared to controls, as evidenced by the observed OTU richness (p=0·0334), the Chao estimate of total OTU richness (p=0·0076), but not the Shannon diversity index (p=0·0821).

Supplementary Figures and Tables

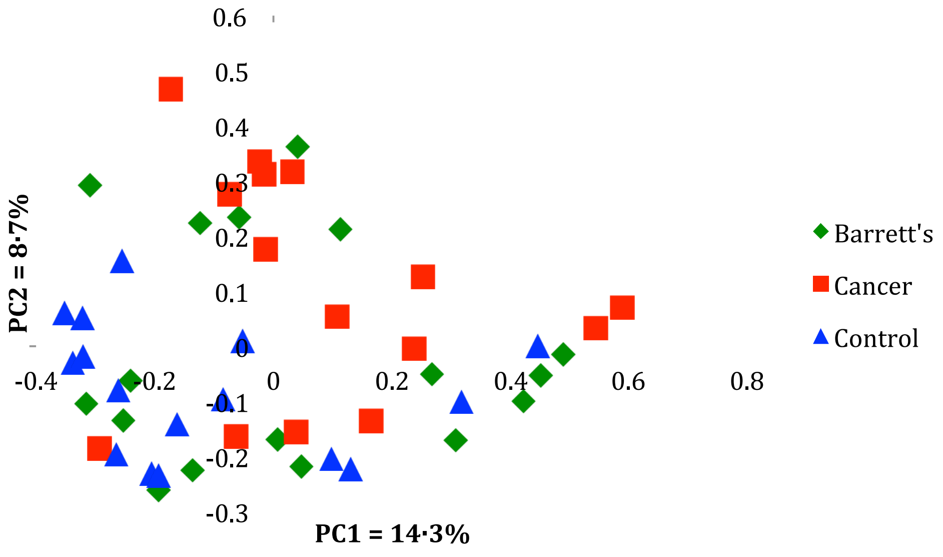

Figure S1. Principal coordinate analysis using the Bray-Curtis algorithm. The PCoA plot shows a significant separation between OAC and control samples (p=0·001). The first axis (PC1) accounts for 14·3% of the sample variance and the second axis (PC2) accounts for 8·7% of the variance.

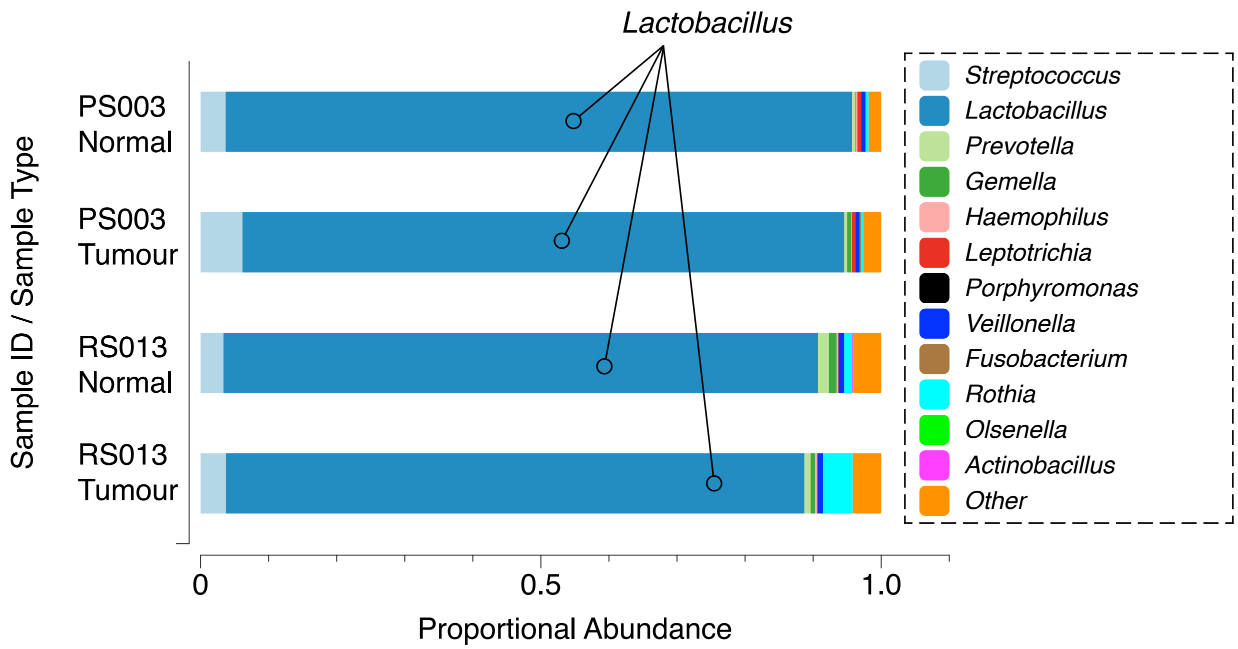

Figure S2. *Lactobacillus* is the dominant taxon in two tumour samples (PS003 and RS013) and matched normal squamous tissue.

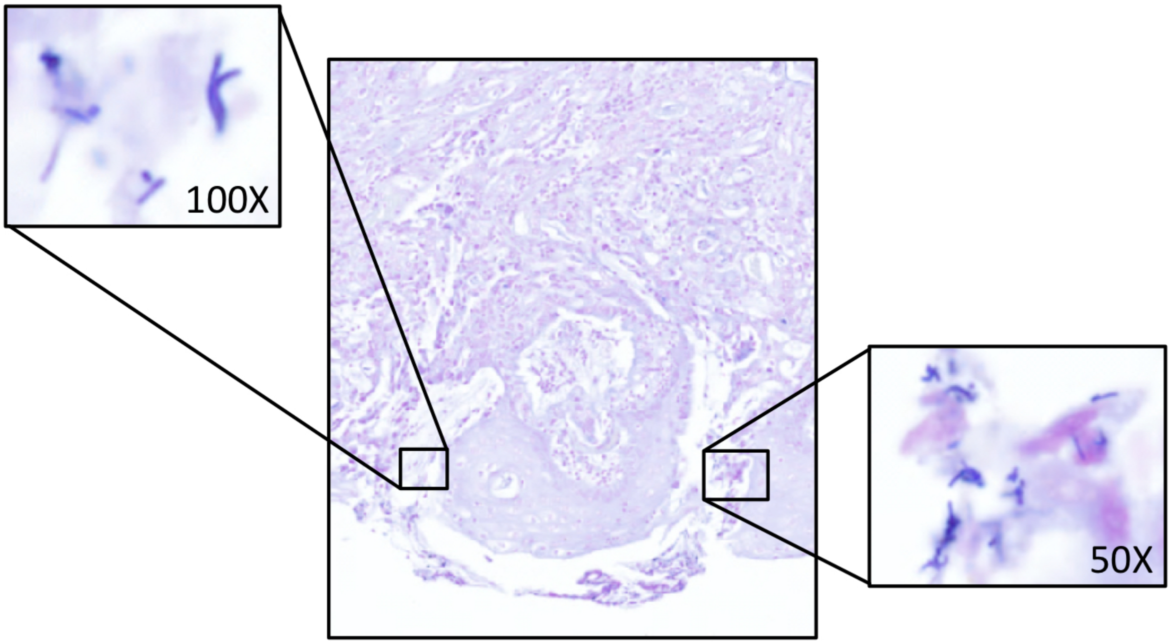

Figure S3. Tumour sample PS003 stained with haematoxylin and eosin, scanned at 20X magnification, imaged at 5% digital zoom. Inserts show Gram stain with Gram-positive rods, imaged at 50% or 100% digital zoom.

Table S1. Clinicopathologic data for patients with oesophageal adenocarcinoma. Patient CTX or RCTX refers to chemotherapy or radio-chemotherapy received at any time during the patient's treatment course. All samples were untreated with chemotherapy (naïve). R1=margin involved, R0=margin clear, RX=information not available.

ID	Patient CTX	Patient Survival (mos)	Sample type	Tumour type	Stage	Pathological TNM	Differentiation
PS/002	Naïve	Alive (70.8)	Surgery	GOJ Type 2	4	T3N2M1	Moderate
PS/003	Naïve	Dead (22.8)	Surgery	GOJ Type 1	3	T3N1M0	Poor
AH/085	CTX	Alive (43.6)	Biopsy	GOJ Type 2	2	T3N0M0	Moderate
ST/041	CTX	Dead (18.6)	Biopsy	GOJ Type 2	3	T4aN2M0	Poor
ST/030	CTX	Alive (38.3)	Surgery	GOJ Type 2	3	T4aN0M0	Moderate
RS/007	CTX	Dead (10.1)	Biopsy	GOJ Type 1	4	T3N3M1	Poor
RS/006	CTX	Dead (11.3)	Biopsy	GOJ Type 2	3	T3N2M0	Poor
SH/071	Naïve	Alive (37.5)	Surgery	GOJ Type 1	3	T3N2M0	Poor
ST/036	RCTX	Dead (4.0)	Biopsy	GOJ Type 3	3	T3N2MX	Poor
ST/037	CTX	Alive (37.6)	Biopsy	GOJ Type 1	2	T3N0M0	Moderate
ED/041	Naïve	Alive (37.7)	Surgery	GOJ Type 3	2	T3N0M0	Moderate
AH/169	Naïve	Alive (30.6)	Surgery	GOJ Type 2	1	T1aN0M0	Moderate
WG008	Naïve	Alive (48.5)	Surgery	GOJ Type 3	2	T3N0M0	Moderate
RS/010	Naïve	Alive (35.2)	Biopsy	GOJ Type 1	1	T1bN0M0	Moderate
AH/135	Naïve	Alive (35.1)	Surgery	GOJ Type 2	1	T1bN0M0	Moderate
RS/013	Naïve	Alive (36.9)	Biopsy	GOJ Type 3	3	T3N3MX	Poor
AH/155	CTX	Alive (34.1)	Biopsy	GOJ Type 1	1	T2N0M0	Poor
RS/029	CTX	Alive (31.9)	Biopsy	GOJ Type 1	1	T2N0M0	Poor
AH/131	RCTX	Dead (19.5)	Biopsy	GOJ Type 2	1	T2N0M0	Moderate

Table S2. Number of patients recruited at each participating UK hospital centre. CAM=Cambridge University Hospitals NHS Foundation Trust, Cambridge; NOT=Nottingham University Hospitals NHS Trust, Nottingham; NEW=Newcastle Upon Tyne Hospital, Newcastle; UCL=University College Hospital London, London; PHH=Hinchingbrooke Hospital, Huntingdon; ED=Edinburgh Royal Infirmary, Edinburgh; RS=Royal Surrey County Hospital National Health Service (NHS) Foundation Trust, Guildford; SH=University of Southampton & University Hospital Southampton NHS Foundation Trust, Southampton; ST=St. Thomas' Hospital, London; and WG=Wigan and Leigh NHS Foundation Trust, Manchester.

Patient group	Centre (n)
Control (n=20)	CAM (15)<br>NOT (5)
Barrett's (n=24)	CAM (16)<br>NEW (2)<br>NOT (2)<br>PHH (1)<br>UCL (3)
Dysplasia (n=23)	CAM (14)<br>NEW (1)<br>NOT (2)<br>UCL (6)
Cancer (n=19)	CAM (7)<br>ED (1)<br>RS (5)<br>SH (1)<br>ST (4)<br>WG (1)

Table S3. Sample accession numbers for 16S rRNA gene amplicon sequencing data deposited in the European Nucleotide Archive.

Sample accession	Patient ID	Sample type	Patient diagnosis	Sample diagnosis	Replicate
ERS418605	BEST2/CAM/0002/R1	Throat swab	Barrett's	-	A
ERS418653	BEST2/CAM/0002/R1	Throat swab	Barrett's	-	B
ERS418684	BEST2/CAM/0002/R1	Cytosponge	Barrett's	-	A
ERS418732	BEST2/CAM/0002/R1	Cytosponge	Barrett's	-	B
ERS473478	BEST2/CAM/0002/R1	Brush	Barrett's	Squamous	A
ERS473526	BEST2/CAM/0002/R1	Brush	Barrett's	Squamous	B
ERS473756	BEST2/CAM/0002/R1	Tissue	Barrett's	Squamous	A
ERS473757	BEST2/CAM/0002/R1	Tissue	Barrett's	Barrett's	A
ERS473804	BEST2/CAM/0002/R1	Tissue	Barrett's	Squamous	B
ERS473805	BEST2/CAM/0002/R1	Tissue	Barrett's	Barrett's	B
ERS418682	BEST2/CAM/0042/R1	Cytosponge	Barrett's	-	A
ERS418730	BEST2/CAM/0042/R1	Cytosponge	Barrett's	-	B
ERS473548	BEST2/CAM/0042/R1	Brush	Barrett's	Squamous	A
ERS473596	BEST2/CAM/0042/R1	Brush	Barrett's	Squamous	B
ERS473664	BEST2/CAM/0042/R1	Tissue	Barrett's	Squamous	-
ERS473665	BEST2/CAM/0042/R1	Tissue	Barrett's	Barrett's	-
ERS418711	BEST2/CAM/0072/R1	Cytosponge	Dysplasia	-	A
ERS418759	BEST2/CAM/0072/R1	Cytosponge	Dysplasia	-	B
ERS418700	BEST2/CAM/0096/R1	Cytosponge	Barrett's	-	A
ERS418748	BEST2/CAM/0096/R1	Cytosponge	Barrett's	-	B
ERS473543	BEST2/CAM/0096/R1	Brush	Barrett's	Squamous	A
ERS473591	BEST2/CAM/0096/R1	Brush	Barrett's	Squamous	B
ERS473656	BEST2/CAM/0096/R1	Tissue	Barrett's	Squamous	-
ERS473657	BEST2/CAM/0096/R1	Tissue	Barrett's	Barrett's	-
ERS418695	BEST2/CAM/0127	Cytosponge	Dysplasia	-	A
ERS418743	BEST2/CAM/0127	Cytosponge	Dysplasia	-	B
ERS418693	BEST2/CAM/0143	Cytosponge	Control	-	A
ERS418741	BEST2/CAM/0143	Cytosponge	Control	-	B
ERS473484	BEST2/CAM/0143	Brush	Control	-	A
ERS473532	BEST2/CAM/0143	Brush	Control	-	B
ERS473655	BEST2/CAM/0143	Tissue	Control	-	-
ERS418696	BEST2/CAM/0153	Cytosponge	Control	-	A
ERS418744	BEST2/CAM/0153	Cytosponge	Control	-	B
ERS473489	BEST2/CAM/0153	Brush	Control	-	A
ERS473537	BEST2/CAM/0153	Brush	Control	-	B
ERS473658	BEST2/CAM/0153	Tissue	Control	-	-
ERS418699	BEST2/CAM/0161	Cytosponge	Control	-	A
ERS418747	BEST2/CAM/0161	Cytosponge	Control	-	B
ERS473542	BEST2/CAM/0161	Brush	Control	-	A
ERS473590	BEST2/CAM/0161	Brush	Control	-	B
ERS473661	BEST2/CAM/0161	Tissue	Control	-	-
ERS418714	BEST2/CAM/0162	Cytosponge	Dysplasia	-	A
ERS418762	BEST2/CAM/0162	Cytosponge	Dysplasia	-	B

ERS418702	BEST2/CAM/0174	Cytosponge	Control	-	A
ERS418750	BEST2/CAM/0174	Cytosponge	Control	-	B
ERS473547	BEST2/CAM/0174	Brush	Control	-	A
ERS473595	BEST2/CAM/0174	Brush	Control	-	B
ERS473666	BEST2/CAM/0174	Tissue	Control	-	-
ERS418704	BEST2/CAM/0197	Cytosponge	Control	-	A
ERS418752	BEST2/CAM/0197	Cytosponge	Control	-	B
ERS473552	BEST2/CAM/0197	Brush	Control	-	-
ERS473669	BEST2/CAM/0197	Tissue	Control	-	A
ERS473717	BEST2/CAM/0197	Tissue	Control	-	B
ERS418707	BEST2/CAM/0202	Cytosponge	Control	-	A
ERS418755	BEST2/CAM/0202	Cytosponge	Control	-	B
ERS473557	BEST2/CAM/0202	Brush	Control	-	-
ERS473672	BEST2/CAM/0202	Tissue	Control	-	A
ERS473720	BEST2/CAM/0202	Tissue	Control	-	B
ERS418710	BEST2/CAM/0208	Cytosponge	Control	-	A
ERS418758	BEST2/CAM/0208	Cytosponge	Control	-	B
ERS473562	BEST2/CAM/0208	Brush	Control	-	-
ERS473677	BEST2/CAM/0208	Tissue	Control	-	A
ERS473725	BEST2/CAM/0208	Tissue	Control	-	B
ERS418712	BEST2/CAM/0216	Cytosponge	Control	-	A
ERS418760	BEST2/CAM/0216	Cytosponge	Control	-	B
ERS473565	BEST2/CAM/0216	Brush	Control	-	A
ERS473613	BEST2/CAM/0216	Brush	Control	-	B
ERS473680	BEST2/CAM/0216	Tissue	Control	-	A
ERS473728	BEST2/CAM/0216	Tissue	Control	-	B
ERS418621	BEST2/CAM/0231	Cytosponge	Dysplasia	-	A
ERS418669	BEST2/CAM/0231	Cytosponge	Dysplasia	-	B
ERS418604	BEST2/CAM/0232/R1	Throat swab	Control	-	A
ERS418627	BEST2/CAM/0232/R1	Cytosponge	Control	-	A
ERS418652	BEST2/CAM/0232/R1	Throat swab	Control	-	B
ERS418675	BEST2/CAM/0232/R1	Cytosponge	Control	-	B
ERS418694	BEST2/CAM/0248	Cytosponge	Barrett's	-	A
ERS418742	BEST2/CAM/0248	Cytosponge	Barrett's	-	B
ERS473485	BEST2/CAM/0248	Brush	Barrett's	Squamous	A
ERS473533	BEST2/CAM/0248	Brush	Barrett's	Squamous	B
ERS473659	BEST2/CAM/0248	Tissue	Barrett's	Squamous	-
ERS473660	BEST2/CAM/0248	Tissue	Barrett's	Barrett's	-
ERS418697	BEST2/CAM/0249	Cytosponge	Barrett's	-	A
ERS418745	BEST2/CAM/0249	Cytosponge	Barrett's	-	B
ERS473490	BEST2/CAM/0249	Brush	Barrett's	Squamous	A
ERS473538	BEST2/CAM/0249	Brush	Barrett's	Squamous	B
ERS473662	BEST2/CAM/0249	Tissue	Barrett's	Squamous	-
ERS473663	BEST2/CAM/0249	Tissue	Barrett's	Barrett's	-
ERS418706	BEST2/CAM/0287	Cytosponge	Dysplasia	-	A
ERS418754	BEST2/CAM/0287	Cytosponge	Dysplasia	-	B

ERS418681	BEST2/CAM/0416/R1	Cytosponge	Dysplasia	-	A
ERS418729	BEST2/CAM/0416/R1	Cytosponge	Dysplasia	-	B
ERS418584	BEST2/CAM/0424	Throat swab	Control	-	A
ERS418613	BEST2/CAM/0424	Cytosponge	Control	-	A
ERS418632	BEST2/CAM/0424	Throat swab	Control	-	B
ERS418661	BEST2/CAM/0424	Cytosponge	Control	-	B
ERS473452	BEST2/CAM/0424	Brush	Control	-	A
ERS473500	BEST2/CAM/0424	Brush	Control	-	B
ERS473750	BEST2/CAM/0424	Tissue	Control	-	A
ERS473798	BEST2/CAM/0424	Tissue	Control	-	B
ERS418587	BEST2/CAM/0425	Throat swab	Control	-	A
ERS418616	BEST2/CAM/0425	Cytosponge	Control	-	A
ERS418635	BEST2/CAM/0425	Throat swab	Control	-	B
ERS418664	BEST2/CAM/0425	Cytosponge	Control	-	B
ERS473462	BEST2/CAM/0425	Brush	Control	-	A
ERS473510	BEST2/CAM/0425	Brush	Control	-	B
ERS473753	BEST2/CAM/0425	Tissue	Control	-	A
ERS473801	BEST2/CAM/0425	Tissue	Control	-	B
ERS418585	BEST2/CAM/0427	Throat swab	Barrett's	-	A
ERS418614	BEST2/CAM/0427	Cytosponge	Barrett's	-	A
ERS418633	BEST2/CAM/0427	Throat swab	Barrett's	-	B
ERS418662	BEST2/CAM/0427	Cytosponge	Barrett's	-	B
ERS473453	BEST2/CAM/0427	Brush	Barrett's	Squamous	A
ERS473501	BEST2/CAM/0427	Brush	Barrett's	Squamous	B
ERS473686	BEST2/CAM/0427	Tissue	Barrett's	Squamous	A
ERS473687	BEST2/CAM/0427	Tissue	Barrett's	Barrett's	A
ERS473734	BEST2/CAM/0427	Tissue	Barrett's	Squamous	B
ERS473735	BEST2/CAM/0427	Tissue	Barrett's	Barrett's	B
ERS418607	BEST2/CAM/0429	Throat swab	Barrett's	-	A
ERS418655	BEST2/CAM/0429	Throat swab	Barrett's	-	B
ERS418686	BEST2/CAM/0429	Cytosponge	Barrett's	-	A
ERS418734	BEST2/CAM/0429	Cytosponge	Barrett's	-	B
ERS473479	BEST2/CAM/0429	Brush	Barrett's	Squamous	A
ERS473527	BEST2/CAM/0429	Brush	Barrett's	Squamous	B
ERS473759	BEST2/CAM/0429	Tissue	Barrett's	Squamous	A
ERS473760	BEST2/CAM/0429	Tissue	Barrett's	Barrett's	A
ERS473807	BEST2/CAM/0429	Tissue	Barrett's	Squamous	B
ERS473808	BEST2/CAM/0429	Tissue	Barrett's	Barrett's	B
ERS418615	BEST2/CAM/0430	Cytosponge	Dysplasia	-	A
ERS418663	BEST2/CAM/0430	Cytosponge	Dysplasia	-	B
ERS418588	BEST2/CAM/0437	Throat swab	Barrett's	-	A
ERS418617	BEST2/CAM/0437	Cytosponge	Barrett's	-	A
ERS418636	BEST2/CAM/0437	Throat swab	Barrett's	-	B
ERS418665	BEST2/CAM/0437	Cytosponge	Barrett's	-	B
ERS473458	BEST2/CAM/0437	Brush	Barrett's	Squamous	A
ERS473506	BEST2/CAM/0437	Brush	Barrett's	Squamous	B

ERS473689	BEST2/CAM/0437	Tissue	Barrett's	Squamous	A
ERS473690	BEST2/CAM/0437	Tissue	Barrett's	Barrett's	A
ERS473737	BEST2/CAM/0437	Tissue	Barrett's	Squamous	B
ERS473738	BEST2/CAM/0437	Tissue	Barrett's	Barrett's	B
ERS418685	BEST2/CAM/0444	Cytosponge	Dysplasia	-	A
ERS418733	BEST2/CAM/0444	Cytosponge	Dysplasia	-	B
ERS418609	BEST2/CAM/0445	Throat swab	Barrett's	-	A
ERS418657	BEST2/CAM/0445	Throat swab	Barrett's	-	B
ERS418688	BEST2/CAM/0445	Cytosponge	Barrett's	-	A
ERS418736	BEST2/CAM/0445	Cytosponge	Barrett's	-	B
ERS473762	BEST2/CAM/0445	Tissue	Barrett's	Squamous	A
ERS473763	BEST2/CAM/0445	Tissue	Barrett's	Barrett's	A
ERS473810	BEST2/CAM/0445	Tissue	Barrett's	Squamous	B
ERS473811	BEST2/CAM/0445	Tissue	Barrett's	Barrett's	B
ERS418591	BEST2/CAM/0446	Throat swab	Barrett's	-	A
ERS418620	BEST2/CAM/0446	Cytosponge	Barrett's	-	A
ERS418639	BEST2/CAM/0446	Throat swab	Barrett's	-	B
ERS418668	BEST2/CAM/0446	Cytosponge	Barrett's	-	B
ERS473463	BEST2/CAM/0446	Brush	Barrett's	Squamous	A
ERS473511	BEST2/CAM/0446	Brush	Barrett's	Squamous	B
ERS473692	BEST2/CAM/0446	Tissue	Barrett's	Squamous	A
ERS473740	BEST2/CAM/0446	Tissue	Barrett's	Squamous	B
ERS473741	BEST2/CAM/0446	Tissue	Barrett's	Barrett's	A
ERS473789	BEST2/CAM/0446	Tissue	Barrett's	Barrett's	B
ERS418687	BEST2/CAM/0448	Cytosponge	Dysplasia	-	A
ERS418735	BEST2/CAM/0448	Cytosponge	Dysplasia	-	B
ERS418593	BEST2/CAM/0449	Throat swab	Barrett's	-	A
ERS418623	BEST2/CAM/0449	Cytosponge	Barrett's	-	A
ERS418641	BEST2/CAM/0449	Throat swab	Barrett's	-	B
ERS418671	BEST2/CAM/0449	Cytosponge	Barrett's	-	B
ERS473466	BEST2/CAM/0449	Brush	Barrett's	Squamous	A
ERS473514	BEST2/CAM/0449	Brush	Barrett's	Squamous	B
ERS473743	BEST2/CAM/0449	Tissue	Barrett's	Squamous	A
ERS473744	BEST2/CAM/0449	Tissue	Barrett's	Barrett's	A
ERS473791	BEST2/CAM/0449	Tissue	Barrett's	Squamous	B
ERS473792	BEST2/CAM/0449	Tissue	Barrett's	Barrett's	B
ERS418590	BEST2/CAM/0451	Throat swab	Control	-	A
ERS418638	BEST2/CAM/0451	Throat swab	Control	-	B
ERS418683	BEST2/CAM/0451	Cytosponge	Control	-	A
ERS418731	BEST2/CAM/0451	Cytosponge	Control	-	B
ERS473465	BEST2/CAM/0451	Brush	Control	-	A
ERS473513	BEST2/CAM/0451	Brush	Control	-	B
ERS473758	BEST2/CAM/0451	Tissue	Control	-	A
ERS473806	BEST2/CAM/0451	Tissue	Control	-	B
ERS418631	BEST2/CAM/0453	Cytosponge	Dysplasia	-	A
ERS418679	BEST2/CAM/0453	Cytosponge	Dysplasia	-	B

ERS418595	BEST2/CAM/0454	Throat swab	Barrett's	-	A
ERS418625	BEST2/CAM/0454	Cytosponge	Barrett's	-	A
ERS418643	BEST2/CAM/0454	Throat swab	Barrett's	-	B
ERS418673	BEST2/CAM/0454	Cytosponge	Barrett's	-	B
ERS473469	BEST2/CAM/0454	Brush	Barrett's	Squamous	A
ERS473517	BEST2/CAM/0454	Brush	Barrett's	Squamous	B
ERS473745	BEST2/CAM/0454	Tissue	Barrett's	Squamous	A
ERS473746	BEST2/CAM/0454	Tissue	Barrett's	Barrett's	A
ERS473793	BEST2/CAM/0454	Tissue	Barrett's	Squamous	B
ERS473794	BEST2/CAM/0454	Tissue	Barrett's	Barrett's	B
ERS418611	BEST2/CAM/0456	Throat swab	Barrett's	-	A
ERS418659	BEST2/CAM/0456	Throat swab	Barrett's	-	B
ERS418690	BEST2/CAM/0456	Cytosponge	Barrett's	-	A
ERS418738	BEST2/CAM/0456	Cytosponge	Barrett's	-	B
ERS473481	BEST2/CAM/0456	Brush	Barrett's	Squamous	A
ERS473529	BEST2/CAM/0456	Brush	Barrett's	Squamous	B
ERS473765	BEST2/CAM/0456	Tissue	Barrett's	Squamous	A
ERS473766	BEST2/CAM/0456	Tissue	Barrett's	Barrett's	A
ERS473813	BEST2/CAM/0456	Tissue	Barrett's	Squamous	B
ERS473814	BEST2/CAM/0456	Tissue	Barrett's	Barrett's	B
ERS418612	BEST2/CAM/0458	Throat swab	Barrett's	-	A
ERS418660	BEST2/CAM/0458	Throat swab	Barrett's	-	B
ERS418692	BEST2/CAM/0458	Cytosponge	Barrett's	-	A
ERS418740	BEST2/CAM/0458	Cytosponge	Barrett's	-	B
ERS473482	BEST2/CAM/0458	Brush	Barrett's	Squamous	A
ERS473530	BEST2/CAM/0458	Brush	Barrett's	Squamous	B
ERS473768	BEST2/CAM/0458	Tissue	Barrett's	Squamous	A
ERS473769	BEST2/CAM/0458	Tissue	Barrett's	Barrett's	A
ERS473816	BEST2/CAM/0458	Tissue	Barrett's	Squamous	B
ERS473817	BEST2/CAM/0458	Tissue	Barrett's	Barrett's	B
ERS418598	BEST2/CAM/0459	Throat swab	Barrett's	-	A
ERS418628	BEST2/CAM/0459	Cytosponge	Barrett's	-	A
ERS418646	BEST2/CAM/0459	Throat swab	Barrett's	-	B
ERS418676	BEST2/CAM/0459	Cytosponge	Barrett's	-	B
ERS473472	BEST2/CAM/0459	Brush	Barrett's	Squamous	A
ERS473520	BEST2/CAM/0459	Brush	Barrett's	Squamous	B
ERS473748	BEST2/CAM/0459	Tissue	Barrett's	Squamous	A
ERS473749	BEST2/CAM/0459	Tissue	Barrett's	Barrett's	A
ERS473796	BEST2/CAM/0459	Tissue	Barrett's	Squamous	B
ERS473797	BEST2/CAM/0459	Tissue	Barrett's	Barrett's	B
ERS418594	BEST2/CAM/0460	Throat swab	Control	-	A
ERS418622	BEST2/CAM/0460	Cytosponge	Control	-	A
ERS418642	BEST2/CAM/0460	Throat swab	Control	-	B
ERS418670	BEST2/CAM/0460	Cytosponge	Control	-	B
ERS473468	BEST2/CAM/0460	Brush	Control	-	A
ERS473516	BEST2/CAM/0460	Brush	Control	-	B

ERS473761	BEST2/CAM/0460	Tissue	Control	-	A
ERS473809	BEST2/CAM/0460	Tissue	Control	-	B
ERS418618	BEST2/CAM/0462	Cytosponge	Dysplasia	-	A
ERS418666	BEST2/CAM/0462	Cytosponge	Dysplasia	-	B
ERS418597	BEST2/CAM/0464	Throat swab	Control	-	A
ERS418619	BEST2/CAM/0464	Cytosponge	Control	-	A
ERS418645	BEST2/CAM/0464	Throat swab	Control	-	B
ERS418667	BEST2/CAM/0464	Cytosponge	Control	-	B
ERS473471	BEST2/CAM/0464	Brush	Control	-	A
ERS473519	BEST2/CAM/0464	Brush	Control	-	B
ERS473764	BEST2/CAM/0464	Tissue	Control	-	A
ERS473812	BEST2/CAM/0464	Tissue	Control	-	B
ERS418689	BEST2/CAM/0466	Cytosponge	Dysplasia	-	A
ERS418737	BEST2/CAM/0466	Cytosponge	Dysplasia	-	B
ERS418601	BEST2/CAM/0470	Throat swab	Barrett's	-	A
ERS418630	BEST2/CAM/0470	Cytosponge	Barrett's	-	A
ERS418649	BEST2/CAM/0470	Throat swab	Barrett's	-	B
ERS418678	BEST2/CAM/0470	Cytosponge	Barrett's	-	B
ERS473474	BEST2/CAM/0470	Brush	Barrett's	Squamous	A
ERS473522	BEST2/CAM/0470	Brush	Barrett's	Squamous	B
ERS473751	BEST2/CAM/0470	Tissue	Barrett's	Squamous	A
ERS473752	BEST2/CAM/0470	Tissue	Barrett's	Barrett's	A
ERS473799	BEST2/CAM/0470	Tissue	Barrett's	Squamous	B
ERS473800	BEST2/CAM/0470	Tissue	Barrett's	Barrett's	B
ERS418629	BEST2/CAM/0472	Cytosponge	Dysplasia	-	A
ERS418677	BEST2/CAM/0472	Cytosponge	Dysplasia	-	B
ERS418691	BEST2/CAM/0475	Cytosponge	Dysplasia	-	A
ERS418739	BEST2/CAM/0475	Cytosponge	Dysplasia	-	B
ERS418600	BEST2/CAM/0487	Throat swab	Control	-	A
ERS418624	BEST2/CAM/0487	Cytosponge	Control	-	A
ERS418648	BEST2/CAM/0487	Throat swab	Control	-	B
ERS418672	BEST2/CAM/0487	Cytosponge	Control	-	B
ERS473457	BEST2/CAM/0487	Brush	Control	-	A
ERS473505	BEST2/CAM/0487	Brush	Control	-	B
ERS473767	BEST2/CAM/0487	Tissue	Control	-	A
ERS473815	BEST2/CAM/0487	Tissue	Control	-	B
ERS473449	BEST2/NEW/0011	Cytosponge	Dysplasia	-	A
ERS473497	BEST2/NEW/0011	Cytosponge	Dysplasia	-	B
ERS418705	BEST2/NEW/0047	Cytosponge	Barrett's	-	A
ERS418753	BEST2/NEW/0047	Cytosponge	Barrett's	-	B
ERS473553	BEST2/NEW/0047	Brush	Barrett's	Squamous	-
ERS473667	BEST2/NEW/0047	Tissue	Barrett's	Squamous	-
ERS473668	BEST2/NEW/0047	Tissue	Barrett's	Barrett's	-
ERS418708	BEST2/NEW/0048	Cytosponge	Barrett's	-	A
ERS418756	BEST2/NEW/0048	Cytosponge	Barrett's	-	B
ERS473558	BEST2/NEW/0048	Brush	Barrett's	Squamous	-

ERS473670	BEST2/NEW/0048	Tissue	Barrett's	Squamous	A
ERS473671	BEST2/NEW/0048	Tissue	Barrett's	Barrett's	A
ERS473718	BEST2/NEW/0048	Tissue	Barrett's	Squamous	B
ERS473719	BEST2/NEW/0048	Tissue	Barrett's	Barrett's	B
ERS418715	BEST2/NOT/0025	Cytosponge	Control	-	A
ERS418763	BEST2/NOT/0025	Cytosponge	Control	-	B
ERS473570	BEST2/NOT/0025	Brush	Control	-	A
ERS473618	BEST2/NOT/0025	Brush	Control	-	B
ERS473683	BEST2/NOT/0025	Tissue	Control	-	A
ERS473731	BEST2/NOT/0025	Tissue	Control	-	B
ERS418709	BEST2/NOT/0027	Cytosponge	Dysplasia	-	A
ERS418757	BEST2/NOT/0027	Cytosponge	Dysplasia	-	B
ERS418780	BEST2/NOT/0031	Cytosponge	Control	-	A
ERS418783	BEST2/NOT/0031	Cytosponge	Control	-	B
ERS473637	BEST2/NOT/0031	Brush	Control	-	A
ERS473640	BEST2/NOT/0031	Brush	Control	-	B
ERS473688	BEST2/NOT/0031	Tissue	Control	-	A
ERS473736	BEST2/NOT/0031	Tissue	Control	-	B
ERS473444	BEST2/NOT/0032	Cytosponge	Control	-	A
ERS473492	BEST2/NOT/0032	Cytosponge	Control	-	B
ERS473645	BEST2/NOT/0032	Brush	Control	-	A
ERS473691	BEST2/NOT/0032	Tissue	Control	-	B
ERS473739	BEST2/NOT/0032	Tissue	Control	-	-
ERS418701	BEST2/NOT/0036	Cytosponge	Dysplasia	-	A
ERS418749	BEST2/NOT/0036	Cytosponge	Dysplasia	-	B
ERS473447	BEST2/NOT/0039	Cytosponge	Control	-	A
ERS473495	BEST2/NOT/0039	Cytosponge	Control	-	B
ERS473649	BEST2/NOT/0039	Brush	Control	-	-
ERS473742	BEST2/NOT/0039	Tissue	Control	-	A
ERS473790	BEST2/NOT/0039	Tissue	Control	-	B
ERS418713	BEST2/NOT/0040	Cytosponge	Barrett's	-	A
ERS418761	BEST2/NOT/0040	Cytosponge	Barrett's	-	B
ERS473566	BEST2/NOT/0040	Brush	Barrett's	Squamous	A
ERS473614	BEST2/NOT/0040	Brush	Barrett's	Squamous	B
ERS473673	BEST2/NOT/0040	Tissue	Barrett's	Squamous	A
ERS473674	BEST2/NOT/0040	Tissue	Barrett's	Barrett's	A
ERS473721	BEST2/NOT/0040	Tissue	Barrett's	Squamous	B
ERS473722	BEST2/NOT/0040	Tissue	Barrett's	Barrett's	B
ERS418778	BEST2/NOT/0046	Cytosponge	Barrett's	-	A
ERS418781	BEST2/NOT/0046	Cytosponge	Barrett's	-	B
ERS473571	BEST2/NOT/0046	Brush	Barrett's	Squamous	A
ERS473619	BEST2/NOT/0046	Brush	Barrett's	Squamous	B
ERS473675	BEST2/NOT/0046	Tissue	Barrett's	Squamous	A
ERS473676	BEST2/NOT/0046	Tissue	Barrett's	Barrett's	A
ERS473723	BEST2/NOT/0046	Tissue	Barrett's	Squamous	B
ERS473724	BEST2/NOT/0046	Tissue	Barrett's	Barrett's	B

ERS473450	BEST2/NOT/0054	Cytosponge	Control	-	A
ERS473498	BEST2/NOT/0054	Cytosponge	Control	-	B
ERS473652	BEST2/NOT/0054	Brush	Control	-	-
ERS473747	BEST2/NOT/0054	Tissue	Control	-	A
ERS473795	BEST2/NOT/0054	Tissue	Control	-	B
ERS418603	BEST2/PHH/0009	Throat swab	Barrett's	-	A
ERS418651	BEST2/PHH/0009	Throat swab	Barrett's	-	B
ERS418680	BEST2/PHH/0009	Cytosponge	Barrett's	-	A
ERS418728	BEST2/PHH/0009	Cytosponge	Barrett's	-	B
ERS473476	BEST2/PHH/0009	Brush	Barrett's	Squamous	A
ERS473524	BEST2/PHH/0009	Brush	Barrett's	Squamous	B
ERS473754	BEST2/PHH/0009	Tissue	Barrett's	Squamous	A
ERS473755	BEST2/PHH/0009	Tissue	Barrett's	Barrett's	A
ERS473802	BEST2/PHH/0009	Tissue	Barrett's	Squamous	B
ERS473803	BEST2/PHH/0009	Tissue	Barrett's	Barrett's	B
ERS418703	BEST2/UCL/0003/R1	Cytosponge	Dysplasia	-	A
ERS418751	BEST2/UCL/0003/R1	Cytosponge	Dysplasia	-	B
ERS473446	BEST2/UCL/0028/R1	Cytosponge	Dysplasia	-	A
ERS473494	BEST2/UCL/0028/R1	Cytosponge	Dysplasia	-	B
ERS418626	BEST2/UCL/0034/R1	Cytosponge	Dysplasia	-	A
ERS418674	BEST2/UCL/0034/R1	Cytosponge	Dysplasia	-	B
ERS473445	BEST2/UCL/0038/R1	Cytosponge	Barrett's	-	A
ERS473493	BEST2/UCL/0038/R1	Cytosponge	Barrett's	-	B
ERS473646	BEST2/UCL/0038/R1	Brush	Barrett's	Squamous	-
ERS473678	BEST2/UCL/0038/R1	Tissue	Barrett's	Squamous	A
ERS473679	BEST2/UCL/0038/R1	Tissue	Barrett's	Barrett's	A
ERS473726	BEST2/UCL/0038/R1	Tissue	Barrett's	Squamous	B
ERS473727	BEST2/UCL/0038/R1	Tissue	Barrett's	Barrett's	B
ERS418779	BEST2/UCL/0041/R1	Cytosponge	Dysplasia	-	A
ERS418782	BEST2/UCL/0041/R1	Cytosponge	Dysplasia	-	B
ERS473443	BEST2/UCL/0047	Cytosponge	Dysplasia	-	A
ERS473491	BEST2/UCL/0047	Cytosponge	Dysplasia	-	B
ERS418698	BEST2/UCL/0049	Cytosponge	Dysplasia	-	A
ERS418746	BEST2/UCL/0049	Cytosponge	Dysplasia	-	B
ERS473448	BEST2/UCL/0055	Cytosponge	Barrett's	-	A
ERS473496	BEST2/UCL/0055	Cytosponge	Barrett's	-	B
ERS473650	BEST2/UCL/0055	Brush	Barrett's	Squamous	-
ERS473681	BEST2/UCL/0055	Tissue	Barrett's	Squamous	A
ERS473682	BEST2/UCL/0055	Tissue	Barrett's	Barrett's	A
ERS473729	BEST2/UCL/0055	Tissue	Barrett's	Squamous	B
ERS473730	BEST2/UCL/0055	Tissue	Barrett's	Barrett's	B
ERS473451	BEST2/UCL/0058	Cytosponge	Barrett's	-	A
ERS473499	BEST2/UCL/0058	Cytosponge	Barrett's	-	B
ERS473653	BEST2/UCL/0058	Brush	Barrett's	Squamous	-
ERS473684	BEST2/UCL/0058	Tissue	Barrett's	Squamous	A
ERS473685	BEST2/UCL/0058	Tissue	Barrett's	Barrett's	A

ERS473732	BEST2/UCL/0058	Tissue	Barrett's	Squamous	B
ERS473733	BEST2/UCL/0058	Tissue	Barrett's	Barrett's	B
ERS473774	OCCAMS/AH/085	Tissue	Cancer	Squamous	A
ERS473775	OCCAMS/AH/085	Tissue	Cancer	Tumour	A
ERS473826	OCCAMS/AH/085	Tissue	Cancer	Squamous	B
ERS473827	OCCAMS/AH/085	Tissue	Cancer	Tumour	B
ERS473628	OCCAMS/AH/131	Tissue	Cancer	Tumour	A
ERS473634	OCCAMS/AH/131	Tissue	Cancer	Tumour	B
ERS473605	OCCAMS/AH/135	Tissue	Cancer	Squamous	B
ERS473606	OCCAMS/AH/135	Tissue	Cancer	Tumour	B
ERS473783	OCCAMS/AH/135	Tissue	Cancer	Squamous	A
ERS473784	OCCAMS/AH/135	Tissue	Cancer	Tumour	A
ERS473623	OCCAMS/AH/155	Tissue	Cancer	Squamous	A
ERS473624	OCCAMS/AH/155	Tissue	Cancer	Tumour	A
ERS473629	OCCAMS/AH/155	Tissue	Cancer	Squamous	B
ERS473630	OCCAMS/AH/155	Tissue	Cancer	Tumour	B
ERS473599	OCCAMS/AH/169	Tissue	Cancer	Squamous	B
ERS473600	OCCAMS/AH/169	Tissue	Cancer	Tumour	B
ERS473777	OCCAMS/AH/169	Tissue	Cancer	Squamous	A
ERS473778	OCCAMS/AH/169	Tissue	Cancer	Tumour	A
ERS473703	OCCAMS/ED/41	Tissue	Cancer	Squamous	A
ERS473704	OCCAMS/ED/41	Tissue	Cancer	Tumour	A
ERS473715	OCCAMS/ED/41	Tissue	Cancer	Squamous	B
ERS473716	OCCAMS/ED/41	Tissue	Cancer	Tumour	B
ERS473770	OCCAMS/PS/002	Tissue	Cancer	Squamous	A
ERS473771	OCCAMS/PS/002	Tissue	Cancer	Tumour	A
ERS473822	OCCAMS/PS/002	Tissue	Cancer	Squamous	B
ERS473823	OCCAMS/PS/002	Tissue	Cancer	Tumour	B
ERS473772	OCCAMS/PS/003	Tissue	Cancer	Squamous	A
ERS473773	OCCAMS/PS/003	Tissue	Cancer	Tumour	A
ERS473824	OCCAMS/PS/003	Tissue	Cancer	Squamous	B
ERS473825	OCCAMS/PS/003	Tissue	Cancer	Tumour	B
ERS473695	OCCAMS/RS/006	Tissue	Cancer	Squamous	A
ERS473696	OCCAMS/RS/006	Tissue	Cancer	Tumour	A
ERS473707	OCCAMS/RS/006	Tissue	Cancer	Squamous	B
ERS473708	OCCAMS/RS/006	Tissue	Cancer	Tumour	B
ERS473693	OCCAMS/RS/007	Tissue	Cancer	Squamous	A
ERS473694	OCCAMS/RS/007	Tissue	Cancer	Tumour	A
ERS473705	OCCAMS/RS/007	Tissue	Cancer	Squamous	B
ERS473706	OCCAMS/RS/007	Tissue	Cancer	Tumour	B
ERS473603	OCCAMS/RS/010	Tissue	Cancer	Squamous	B
ERS473604	OCCAMS/RS/010	Tissue	Cancer	Tumour	B
ERS473781	OCCAMS/RS/010	Tissue	Cancer	Squamous	A
ERS473782	OCCAMS/RS/010	Tissue	Cancer	Tumour	A
ERS473607	OCCAMS/RS/013	Tissue	Cancer	Squamous	B
ERS473608	OCCAMS/RS/013	Tissue	Cancer	Tumour	B

ERS473785	OCCAMS/RS/013	Tissue	Cancer	Squamous	A
ERS473786	OCCAMS/RS/013	Tissue	Cancer	Tumour	A
ERS473625	OCCAMS/RS/029	Tissue	Cancer	Squamous	A
ERS473626	OCCAMS/RS/029	Tissue	Cancer	Tumour	A
ERS473631	OCCAMS/RS/029	Tissue	Cancer	Squamous	B
ERS473632	OCCAMS/RS/029	Tissue	Cancer	Tumour	B
ERS473697	OCCAMS/SH/071	Tissue	Cancer	Squamous	A
ERS473698	OCCAMS/SH/071	Tissue	Cancer	Tumour	A
ERS473709	OCCAMS/SH/071	Tissue	Cancer	Squamous	B
ERS473710	OCCAMS/SH/071	Tissue	Cancer	Tumour	B
ERS473844	OCCAMS/ST/030	Tissue	Cancer	Squamous	A
ERS473845	OCCAMS/ST/030	Tissue	Cancer	Tumour	A
ERS473847	OCCAMS/ST/030	Tissue	Cancer	Squamous	B
ERS473848	OCCAMS/ST/030	Tissue	Cancer	Tumour	B
ERS473699	OCCAMS/ST/036	Tissue	Cancer	Squamous	A
ERS473700	OCCAMS/ST/036	Tissue	Cancer	Tumour	A
ERS473711	OCCAMS/ST/036	Tissue	Cancer	Squamous	B
ERS473712	OCCAMS/ST/036	Tissue	Cancer	Tumour	B
ERS473701	OCCAMS/ST/037	Tissue	Cancer	Squamous	A
ERS473702	OCCAMS/ST/037	Tissue	Cancer	Tumour	A
ERS473713	OCCAMS/ST/037	Tissue	Cancer	Squamous	B
ERS473714	OCCAMS/ST/037	Tissue	Cancer	Tumour	B
ERS473776	OCCAMS/ST/041	Tissue	Cancer	Squamous	A
ERS473828	OCCAMS/ST/041	Tissue	Cancer	Squamous	B
ERS473843	OCCAMS/ST/041	Tissue	Cancer	Tumour	A
ERS473846	OCCAMS/ST/041	Tissue	Cancer	Tumour	B
ERS473601	OCCAMS/WG/008	Tissue	Cancer	Squamous	B
ERS473602	OCCAMS/WG/008	Tissue	Cancer	Tumour	B
ERS473779	OCCAMS/WG/008	Tissue	Cancer	Squamous	A
ERS473780	OCCAMS/WG/008	Tissue	Cancer	Tumour	A
ERS473579	Neg_control_Brush10	Reagents		-	-
ERS473580	Neg_control_Brush2	Reagents		-	-
ERS473581	Neg_control_Brush3	Reagents		-	-
ERS473582	Neg_control_Brush4	Reagents		-	-
ERS473583	Neg_control_Brush5	Reagents		-	-
ERS473584	Neg_control_Brush6	Reagents		-	-
ERS473829	Neg_control_Brush7	Reagents		-	-
ERS473577	Neg_control_Brush8	Reagents		-	-
ERS473578	Neg_control_Brush9	Reagents		-	-
ERS418722	Neg_control_Cytosponge2	Reagents		-	A
ERS418770	Neg_control_Cytosponge2	Reagents		-	B
ERS418719	Neg_control_Cytosponge21	Reagents		-	A
ERS418767	Neg_control_Cytosponge21	Reagents		-	B
ERS418720	Neg_control_Cytosponge22	Reagents		-	A
ERS418768	Neg_control_Cytosponge22	Reagents		-	B
ERS418721	Neg_control_Cytosponge23	Reagents		-	A

ERS418769	Neg_control_Cytosponge23	Reagents	-	B
ERS418723	Neg_control_Cytosponge3	Reagents	-	A
ERS418771	Neg_control_Cytosponge3	Reagents	-	B
ERS418724	Neg_control_Cytosponge4	Reagents	-	A
ERS418772	Neg_control_Cytosponge4	Reagents	-	B
ERS418725	Neg_control_Cytosponge5	Reagents	-	A
ERS418773	Neg_control_Cytosponge5	Reagents	-	B
ERS418726	Neg_control_Cytosponge6	Reagents	-	A
ERS418774	Neg_control_Cytosponge6	Reagents	-	B
ERS473575	Neg_control_Cytosponge7	Reagents	-	-
ERS473576	Neg_control_Cytosponge8	Reagents	-	-
ERS418716	Neg_control_Throat_swab11	Reagents	-	A
ERS418764	Neg_control_Throat_swab11	Reagents	-	B
ERS418717	Neg_control_Throat_swab12	Reagents	-	A
ERS418765	Neg_control_Throat_swab12	Reagents	-	B
ERS418718	Neg_control_Throat_swab13	Reagents	-	A
ERS418766	Neg_control_Throat_swab13	Reagents	-	B
ERS473830	Neg_control_Tissue14	Reagents	-	-
ERS473831	Neg_control_Tissue15	Reagents	-	-
ERS473832	Neg_control_Tissue16	Reagents	-	-
ERS473833	Neg_control_Tissue17	Reagents	-	-
ERS473834	Neg_control_Tissue18	Reagents	-	-
ERS473835	Neg_control_Tissue19	Reagents	-	-
ERS473836	Neg_control_Tissue20	Reagents	-	-
ERS473837	Neg_control_Tissue24	Reagents	-	-
ERS473609	Neg_control_Tissue25	Reagents	-	A
ERS473787	Neg_control_Tissue25	Reagents	-	B
ERS473610	Neg_control_Tissue26	Reagents	-	A
ERS473788	Neg_control_Tissue26	Reagents	-	B
ERS418727	PCR_water_blank1	Water	-	A
ERS418775	PCR_water_blank1	Water	-	B
ERS418776	PCR_water_blank2	Water	-	A
ERS418777	PCR_water_blank2	Water	-	B
ERS473585	PCR_water_blank3	Water	-	A
ERS473586	PCR_water_blank3	Water	-	B
ERS473635	PCR_water_blank4	Water	-	-
ERS473636	PCR_water_blank5	Water	-	-
ERS473838	PCR_water_blank6	Water	-	A
ERS473839	PCR_water_blank6	Water	-	B
ERS473840	PCR_water_blank6	Water	-	C
ERS473841	PCR_water_blank7	Water	-	-
ERS473842	PCR_water_blank8	Water	-	-
